# Early death from childhood cancer: First medical record‐level analysis reveals insights on diagnostic timing and cause of death

**DOI:** 10.1002/cam4.6609

**Published:** 2023-10-03

**Authors:** Katherine T. Lind, Elizabeth Molina, Amy Mellies, Kami Wolfe Schneider, William Daley, Adam L. Green

**Affiliations:** ^1^ Department of Pediatrics, Center for Cancer and Blood Disorders, Children's Hospital Colorado University of Colorado School of Medicine Aurora Colorado USA; ^2^ Population Health Shared Resource University of Colorado Cancer Center Aurora Colorado USA

**Keywords:** access to care, childhood cancer, disparities, early death, neoplasm, pediatrics

## Abstract

**Background:**

Approximately 7.5% of pediatric cancer deaths occur in the first 30 days post diagnosis, termed early death (ED). Previous database‐level analyses identified increased ED in Black/Hispanic patients, infants, late adolescents, those in poverty, and with specific diagnoses. Socioeconomic and clinical risk factors have never been assessed at the medical record level and are poorly understood.

**Methods:**

We completed a retrospective case–control study of oncology patients diagnosed from 1995 to 2016 at Children's Hospital Colorado. The ED group (*n* = 45) was compared to a non‐early death (NED) group surviving >31 days, randomly selected from the same cohort (*n* = 44). Medical records and death certificates were manually reviewed for sociodemographic and clinical information to identify risk factors for ED.

**Results:**

We identified increased ED risk in central nervous system (CNS) tumors and, specifically, high‐grade glioma and atypical teratoid/rhabdoid tumor. There was prolonged time from symptom onset to seeking care in the ED group (29.4 vs. 9.8 days) with similar time courses to diagnosis thereafter. Cause of death was most commonly from tumor progression in brain/CNS tumors and infection in hematologic malignancies.

**Conclusions:**

In this first medical record‐level analysis of ED, we identified socioeconomic and clinical risk factors. ED was associated with longer time from first symptoms to presentation, suggesting that delayed presentation may be an addressable risk factor. Many individual patient‐level risk factors, including socioeconomic measures and barriers to care, were unable to be assessed through record review, highlighting the need for a prospective study to understand and address childhood cancer ED.

## 1 INTRODUCTION

While childhood cancer outcomes have significantly improved over the last few decades, there remains a group of children with cancer who do not survive long enough to begin treatment or who die early in the treatment process.^1^ This group, the early death (ED) population, represents 7.5% of childhood cancer deaths and is defined as patients who die within one month of their diagnosis.^2^, ^3^ While non‐early death (NED) in childhood cancer is often due to aggressive tumors unresponsive to therapy, ED cases may often be preventable by addressing barriers to care prior to diagnosis and in improvement of care initially after diagnosis.^4^, ^5^, ^6^, ^7^, ^8^, ^9^

The largest study of the ED group was a population‐based study, published in 2017, that utilized the SEER database.^2^ This population‐based study focused on patients 0–19 years of age at diagnosis with cancer from 1992 to 2011 and identified that 7.5% of all cancer deaths were in the ED population. The highest risk of ED was found in patients with acute myeloid leukemia, infantile acute lymphoblastic leukemia, hepatoblastoma, and malignant brain tumors. When dividing the study groups into the categories of hematologic malignancies, central nervous system (CNS) malignancies, and solid tumors outside of the CNS, multivariable analysis identified a significant association with ED in at least two disease groups for patients age less than 1 year or 15–19 years, patients identifying as Black race, and patients of Hispanic ethnicity. Among patients with hematologic malignancies, living in counties with lower than the median average income was associated with ED. In addition, among patients with solid tumors, distant (metastatic) disease at diagnosis was additionally associated with ED.

While this study evaluated ED on a population or database level, there have been no medical record‐level studies of ED thus far. As the SEER database, like most cancer databases, is without patient‐level socioeconomic measures or detailed information regarding cause of death, further work remains to better understand barriers to care, risk for ED, and causes of death. The SEER database, while providing a unique opportunity to review large volumes of patients, does not provide adequate information to fully understand this group of patients.^10^, ^11^, ^12^

Further, this database‐level perspective does not include information such as presenting signs and symptoms, time course of symptom onset to diagnosis and death, or socioeconomic information such as parental employment status, health insurance status prior to diagnosis, access to primary care, etc. Without more detailed patient‐level data, we are unable to design interventions to reduce ED. Information that has not yet been elucidated includes more individualized socioeconomic factors such as parental health literacy, access to a regular primary care physician, family structure and parental employment status, health insurance status prior to diagnosis, access to regular transportation, perceived barriers to diagnosis or therapy, time course from symptom onset to seeking care, presenting symptoms, and cause of death. This information is essential if we are to develop interventions to reduce the incidence of ED, ultimately improving survival of childhood cancer overall. To investigate these factors, we completed a retrospective cohort study of patients treated at Children's Hospital Colorado (CHCO) who suffered ED from childhood cancer. Patients were selected from the institutional hematology, oncology, and bone marrow transplant (HOB) database and then charts were manually reviewed by a pediatric oncology clinical fellow. We hypothesized that there would be more specific patient data available through electronic medical record (EMR) that will help us to better characterize the population of ED patients in pediatric cancer.

## 2 METHODS

### 2.1 Study design

This was a retrospective case–control study using patient level‐based data extracted from the CHCO EMR. We received Colorado Multi‐Institutional Review Board approval (#16‐2050) to complete this work.

### 2.2 Data source and study cohort

**FIGURE 1:**
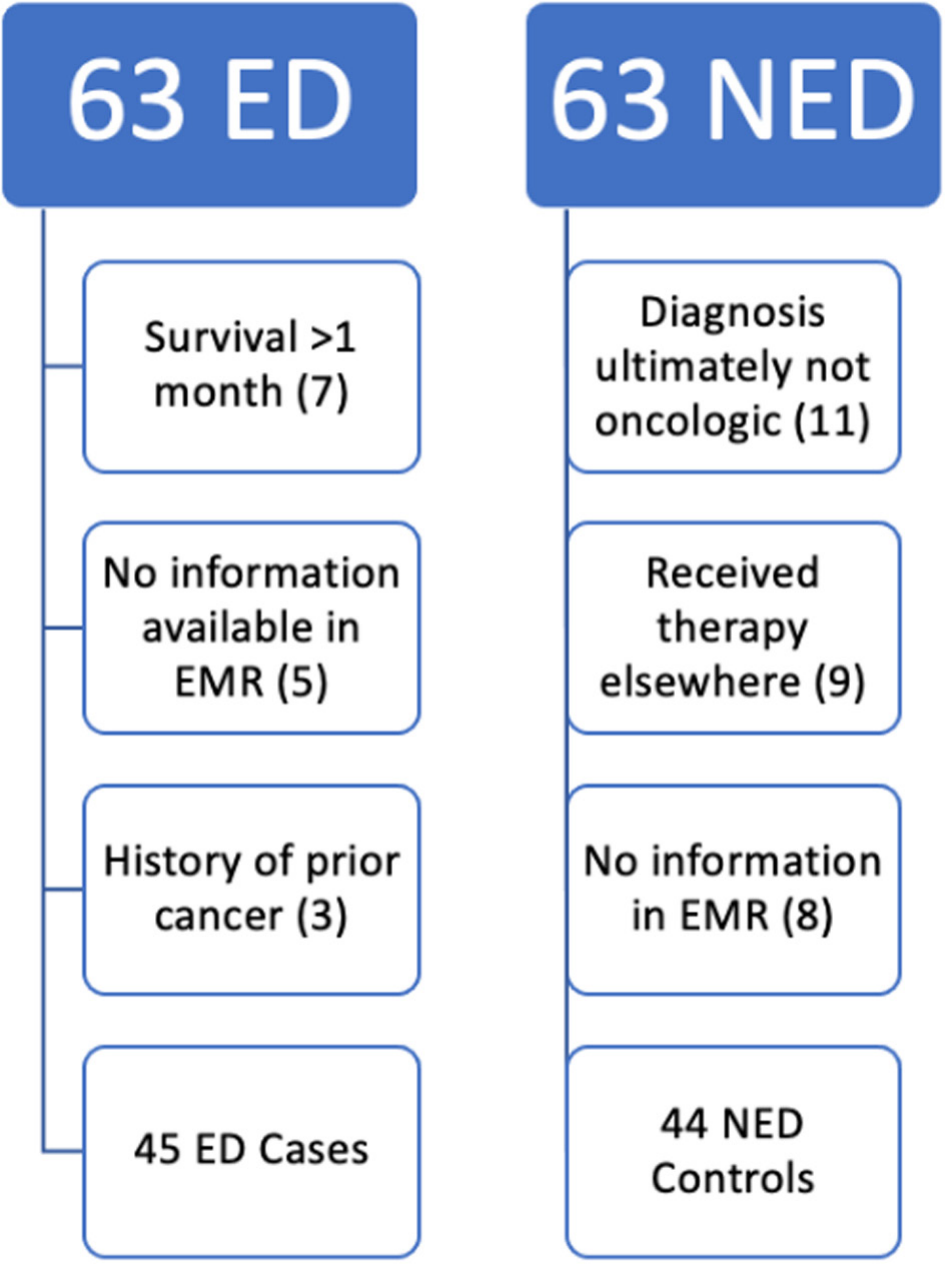
Patient selection process. Patients identified by local database totaled 63 early death patients and 63 non‐early death patients and were subsequently reviewed and excluded for reasons indicated.

Individuals diagnosed from 1995 to 2016 and treated at CHCO were selected from a cohort database comprising of oncology patients at CHCO. Patients who died within 1 month of diagnosis were termed the ED group. The control group (or NED group) included patients who were diagnosed with an oncologic disorder from 1995 to 2016 and survived longer than 30 days. These patients were randomly chosen from the same cohort database comprised of all oncology patients at CHCO. Once the initial patient list was determined, patients were excluded if they had a history of prior cancer, had post‐transplant lymphoproliferative disorder (PTLD), or if their EMR had not been updated to contain their information and notes (see figure 1).

Once the patients were selected, the EMR was manually reviewed for the following information: demographic data including age at diagnosis, primary language, race, ethnicity, address, health insurance, parental employment status; and diagnostic data which include date and nature of first symptoms, date of first seeking care, date of first seeing a specialist, diagnosis (including grade/stage), any genetic evaluation pursued, if the patient received chemotherapy (if so, on which protocol), surgery (if so, procedure and date), or radiation (date and field), date of death, if an autopsy was performed, and cause of death. Death certificates were obtained to identify/compare data on race, ethnicity, and cause of death among cases with that in the EMR, if available. Records, including all relevant primary care notes, emergency room, admission and progress notes, oncology consult notes, surgical procedure notes, discharge summaries, social work notes, and death notes were reviewed manually. This review was completed by the first author, a pediatric hematology/oncology fellow.

To categorize symptoms, initial symptoms were grouped into the following categories: hematologic (including symptoms of anemia such as pallor, extreme fatigue, or thrombocytopenia such as nosebleeds, petechiae, bruising, bleeding, etc.), mass (when the symptom included an identified mass on patient report or exam), B symptoms (to describe weight loss, night sweats, diffuse lymphadenopathy, and fevers), pain, and neurological symptoms (including ataxia, cranial nerve palsies, impaired balance, paresthesias, etc.).

#### 2.2.1 Statistics

##### Patient characteristics

Patient characteristics (age group, sex, race, ethnicity, primary language spoken at home, health insurance status, Colorado residence, rural residence status, year of diagnosis, type of malignancy, grade/stage or risk status [chosen based on disease], and census tract characteristics) were compared using a chi‐squared test, or Fisher's exact test when expected count included less than five patients, for proportions. Odds ratios with a 90% CI were calculated to estimate the odds of ED. With the consideration of the small patient sample in this study, *p* values were evaluated at a significance level of 0.1.

### 2.3 Definitions in census tract data

Census tract characteristics were obtained using patient address and divided based on the median value of characteristics among the study populations. Language isolation was defined as percentage of households in census tract where no member 14 years old and older speaks only English or speaks a non‐English language and speaks English “very well,” with disadvantaged defined as greater than 4.5%.

Foreign born status, derived from census tract data, was defined as percentage of persons in census tract who were foreign born with disadvantaged defined as greater than 6.3%. Educational attainment was defined as percentage of persons 25 years and older without a high school degree or equivalent, with greater than 8.7% meeting criteria for disadvantaged.

Poverty status was defined as persons below 100% poverty level, with criteria for disadvantaged being greater than 9.3%.

## 3 RESULTS

Initially, 63 ED cases and 62 NED controls were selected from the HOB database. Patients were subsequently excluded for the following reasons: diagnosis of PTLD or history of previous cancer diagnosis, survival for greater than 30 days past oncologic diagnosis (in cases), patients who were seen by oncology but ultimately not diagnosed with an oncologic process, patients who were treated at other institutions after diagnosis, and patients who had a lack of any information in EMR. This resulted in *n* = 45 ED and *n* = 44 NED patients (Figure 1). For details regarding individual patient courses, please see Table S2.

### 3.1 Patient characteristics and census tract data

Specific patient characteristics, as described in Table 1, were compared among all malignancies, and then grouped into hematologic malignancies, brain/CNS tumors, and other solid tumors. Comparing all malignancies, groups were largely similar between ED and NED patients, with the *p* values not meeting statistical significance except primary language (English vs. other vs. unknown, *p* = 0.028), grade (low vs. high vs. unknown, *p* < 0.001), and stage (localized vs. disseminated vs. unknown, *p* = 0.035). When comparing odds ratios for these factors among all ED and NED, there was an increased odds of ED for patients with brain tumors (OR 3.25, 90% CI 1.23, 8.57, *p* = 0.046).

**TABLE 1:** Patient and tumor characteristics in ED versus NED.

	All malignancies						
	ED (*N* = 45)		NED (*N* = 44)		Test of proportions^a^	Early death OR (90% CI)	*p*‐Value
	*N*	%	*N*	%	*p*‐Value		
Individual characteristics							
Age					0.246		
<1 year	6	13.33	2	4.55		4.71 (0.99–22.45)	0.102
1–14 years	32	71.11	31	70.45		1.62 (0.66–3.98)	0.375
15–29 years	7	15.56	11	25.00		ref	
Sex					0.730		
Male	26	57.78	27	61.36		ref	
Female	19	42.22	17	38.64		1.16 (0.57–2.36)	0.731
Race					0.112		
White	37	82.22	29	65.91		ref	
Other	5	11.11	13	29.55		0.30 (0.12–0.78)	0.039
Unknown	3	6.67	2	4.55		1.18 (0.25–5.57)	0.864
Ethnicity					1.000		
Non‐Hispanic	28	62.22	28	63.64		ref	
Hispanic	14	31.11	14	31.82		1.00 (0.47–2.14)	1.000
Unknown	3	6.67	2	4.55		1.50 (0.31–7.17)	0.670
Primary language					0.028		
English	30	66.67	37	84.09		ref	
Other	4	8.89	5	11.36		0.99 (0.30–3.20)	0.985
Unknown	11	24.44	2	4.55		6.78 (1.80–25.57)	0.018
Health insurance					0.132		
Private (only)	14	31.11	19	43.18		ref	
Public (any)/uninsured	13	28.89	16	36.36		1.10 (0.47–2.57)	0.849
Unknown	18	40.00	9	20.45		2.71 (1.12–6.59)	0.064
Colorado residence					0.547		
Yes	38	84.44	35	79.55		ref	
No/unknown	7	15.56	9	20.45		0.72 (0.29–1.79)	0.548
Rural residence					0.192		
Urban	41	91.11	35	79.55		ref	
Rural	3	6.67	8	18.18		0.32 (0.10–1.04)	0.111
Unknown	1	2.22	1	2.27		0.85 (0.08–9.01)	0.912
Year of diagnosis					0.944		
1995–2000	6	13.33	5	11.36		ref	
2001–2005	12	26.67	12	27.27		0.83 (0.25–2.77)	0.803
2006–2010	16	35.56	14	31.82		0.95 (0.30–3.05)	0.945
2011–2016	11	24.44	13	29.55		0.71 (0.21–2.35)	0.633
Type of malignancy					0.124		
Solid	6	13.33	13	29.55		ref	
Brain/CNS	24	53.33	16	36.36		3.25 (1.23–8.57)	0.046
Hematologic	15	33.33	15	34.09		2.17 (0.79–5.95)	0.208
Grade					<0.001		
Low	1	2.22	12	27.27		ref	
High	16	35.56	4	9.09		48.00 (6.87–335.08)	0.001
Unknown	28	62.22	28	63.64		12.00 (2.05–70.27)	0.021
Stage					0.035		
Localized	0	0.00	6	13.64		ref	
Disseminated	7	15.56	7	15.91		*^*	^
Unknown	38	84.44	31	70.45		*^*	^
Risk					0.893		
Standard risk	5	11.11	5	11.36		ref	
High risk	10	22.22	8	18.18		1.25 (0.34–4.59)	0.778
Unknown	30	66.67	31	70.45		0.97 (0.32–2.97)	0.962
Census tract characteristics^b^							
Language isolation^c^							
Advantaged	21	46.67	24	54.55	0.759	ref	
Disadvantaged	23	51.11	19	43.18		1.38 (0.68–2.81)	0.451
Unknown	1	2.22	1	2.27		1.14 (0.11–12.32)	0.926
Foreign born^d^					0.694		
Advantaged	20	44.44	24	54.55		ref	
Disadvantaged	24	53.33	19	43.18		1.52 (0.75–3.08)	0.335
Unknown	1	2.22	1	2.27		1.20 (0.11–12.95)	0.9
Educational attainment^e^					0.833		
Advantaged	23	51.11	20	45.45		ref	
Disadvantaged	21	46.67	23	52.27		0.79 (0.39–1.61)	0.592
Unknown	1	2.22	1	2.27		0.87 (0.08–9.40)	0.923
Poverty^f^					1.000		
Advantaged	22	48.89	22	50.00		ref	
Disadvantaged	22	48.89	21	47.73		1.05 (0.52–2.12)	0.914
Unknown	1	2.22	1	2.27		1.00 (0.09–10.79)	1

*Note*: ^, OR not calculated due to 1 or more empty cells.

Abbreviations: CNS, central nervous system; ED, early death; NED, non‐early death.

^a^ Chi‐square test (or Fisher's exact test when expected count includes cells <5) for proportions.

^b^ Census tract derived from patient address and divided based on the median value of characteristic among the study population.

^c^ Percentage of households in census tract where no member 14 years old and over^1^ speaks only English or^2^ speaks a non‐English language and speaks English “very well.” Disadvantaged: >4.5%.

^d^ Percentage of persons in census tract who are foreign born. Disadvantaged: >6.3%.

^e^ Percentage of persons 25 years and older without a high school degree or equivalent. Disadvantaged: >8.7%.

^f^ Percentage of persons below 100% poverty level. Disadvantaged: >9.3%.

Within the brain/CNS tumor group, there was a significant difference comparing high versus low grade (*p* < 0.001) which corresponded to an increased odds of ED for high‐grade tumors versus low‐grade tumors (OR 48.00, 90% CI 6.87, 335.08, *p* = 0.001) and to unknown tumor grade versus low grade (OR 12.00, 90% CI 2.05, 70.27, *p* = 0.021). Significant differences in patient characteristics or increased odds ratio of ED based on patient characteristics or census tract data was not otherwise observed. Of those cases under 1 year of age, none underwent genetic sequencing evaluation for cancer predisposition syndromes.

Patient‐level characteristics within malignancy group (hematologic malignancies, solid tumors, and brain/CNS tumors) were also reviewed (Table S1).

#### 3.1.1 Diagnoses

Disease groups were further subcategorized (Table 2). Within brain/CNS tumors, high‐grade gliomas conferred 15.2 times greater risk of ED (90% CI 2.549, 336.850 *p* = 0.0019), and atypical teratoid/rhabdoid tumors conferred 5.48 times greater risk of ED (90% CI 1.306, ∞, *p* = 0.0610). On the contrary, low‐grade gliomas had a decreased odds of ED (OR 0.104, 90% CI 0.005, 0.670, *p* = 0.0273). There were no additional statistically significant differences between ED and NED groups by diagnostic subgroup, although it is notable that there were five patients with AML in the ED cohort as compared to one patient with AML in the NED group.

**TABLE 2:** Specific disease category of cases versus controls.

Disease category	ED (*n* = 45)		NED (*n* = 44)		Early death OR (90% CI)	*p*‐Value
	*N*	%	*N*	%		
Brain/CNS tumors						
Medulloblastoma	4	8.89	5	11.36	0.763 (0.181–3.050)	0.9710
Atypical teratoid/rhabdoid tumor	4	8.89	0	0	5.477 (1.306–∞)	0.0610
Brain/CNS other	1	2.22	1	2.27	0.978 (0.025–38.472)	1.0000
Choroid plexus carcinoma	2	4.44	0	0	2.398 (0.455–∞)	0.2528
High‐grade glioma	12	26.67	1	2.27	15.255 (2.549–336.850)	0.0019
Low‐grade glioma	1	2.22	8	18.18	0.104 (0.005–0.670)	0.0273
Hematologic malignancies						
ALL	9	20.00	9	20.45	0.973 (0.358–2.641)	1.0000
AML	5	11.11	1	2.27	5.289 (0.721–127.996)	0.2131
Hodgkin's lymphoma	0	0.00	2	4.55	0.398 (0–2.098)	0.2416
Non‐Hodgkin's lymphoma	1	2.22	3	6.82	0.314 (0.012–2.970)	0.5994
Solid tumors						
Wilms tumor	0	0.00	3	6.82	0.245 (0–1.104)	0.1166
Neuroblastoma/related	1	2.22	3	6.82	0.314 (0.012–2.970)	0.5994
Sarcoma (bone)	1	2.22	2	4.55	0.481 (0.016–6.349)	0.9830
Sarcoma (soft tissue)	1	2.22	4	9.09	0.227 (0.035–1.480)	0.3467
Solid tumor (other)	3	6.67	2	4.55	1.493 (0.221–12.574)	1.0000

Abbreviations: ALL, acute lymphoblastic leukemia; AML, acute myeloblastic leukemia; CNS, central nervous system; ED, early death; NED, non‐early death.

#### 3.1.2 Symptoms at diagnosis

**FIGURE 2:**
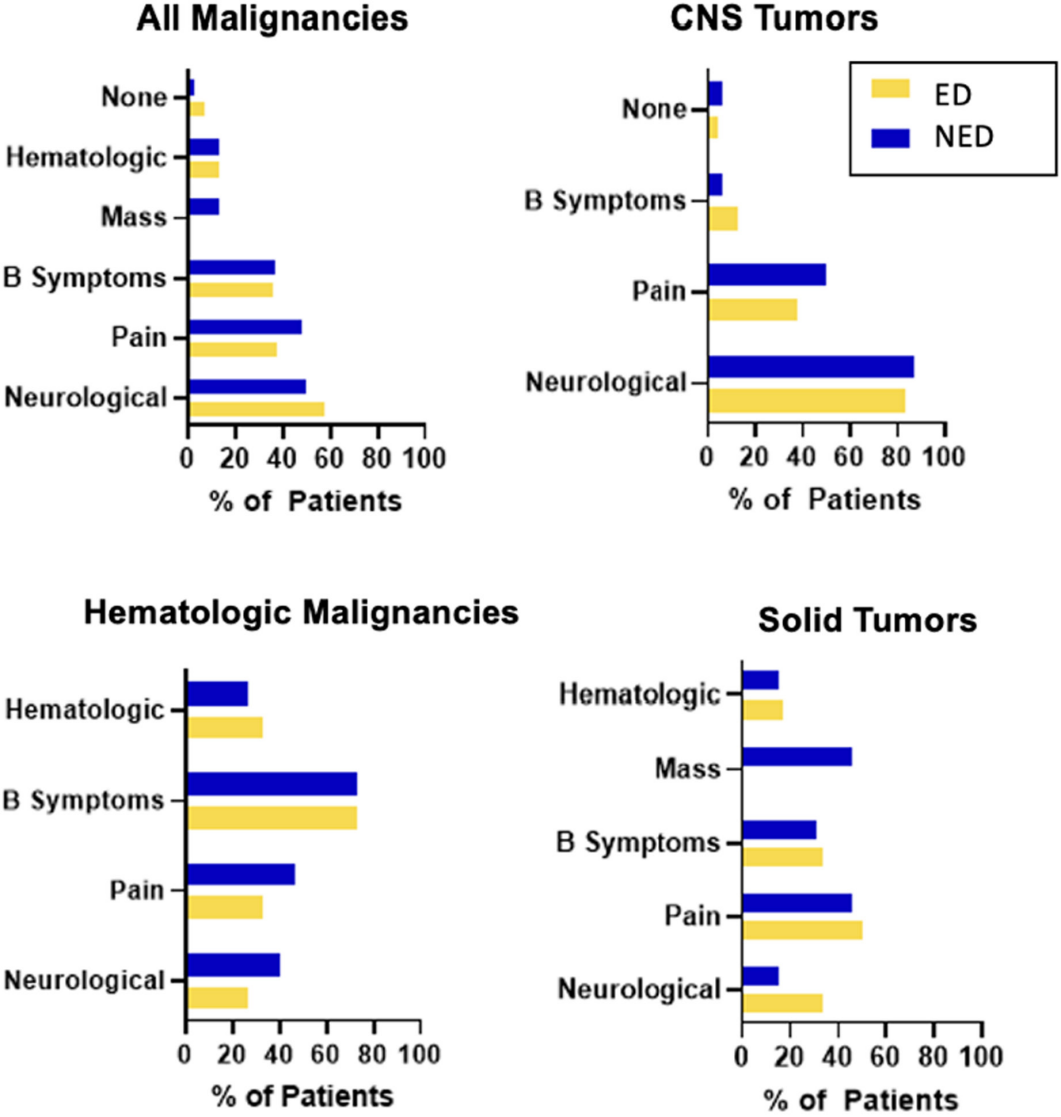
Symptoms at diagnosis. Presenting symptoms of patients from all diseases and subdivided by disease type. Central nervous system tumors included malignancies of the brain and spinal cord, hematologic malignancies included leukemia and lymphoma patients, and solid tumors encompassed all others. Hematologic symptoms included cytopenia, petechiae, epistaxis, and excessive bruising or other bleeding. Mass was used to describe a mass found on exam by either patient, family, or provider. B symptoms included fevers, weight loss, night sweats, and diffuse lymphadenopathy. Neurological symptoms incorporated any neurological symptoms or changes including ataxia, cranial nerve palsies, impaired balance, paresthesias, etc.

Presenting symptoms leading to diagnosis were categorized into hematologic (including bruising, prolonged epistaxis, pallor with anemia), mass (as noted on physical exam), B symptoms (fevers, weight loss, night sweats, generalized lymphadenopathy), pain, and neurological (including changes such as ataxia, weakness, or vision changes). There were no significant differences in each group when comparing all ED to all NED cases, nor when ED and NED groups were compared within disease groups (CNS malignancies, hematologic malignancies, and solid tumors) (Figure 2).

#### 3.1.3 Treatment

**FIGURE 3:**
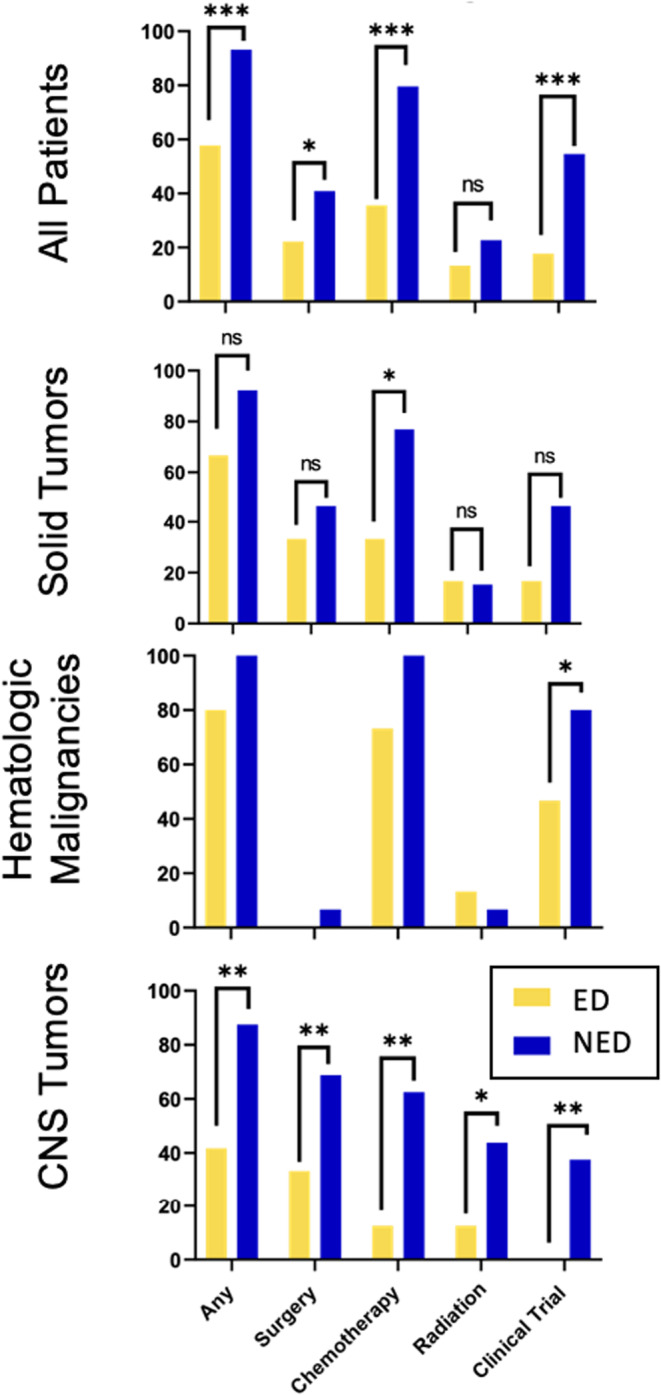
Treatment of early death versus non‐early death group. The x‐axis represents percentage of patients within each group and the y‐axis delineates any type of treatment and then is broken down to include surgery, chemotherapy, radiation, and enrollment on a clinical trial. Statistical significance is marked as * for *p* < 0.1, ** for *p* < 0.05 and ** for *p* < 0.001.

Significant differences were noted between ED and NED patients regarding treatment received, as described in Figure 3. When comparing all ED and NED patients, those who suffered ED were significantly less likely to receive any treatment at all (OR 9.99, 90% CI 3.32–30.06, *p* < 0.001). When looking at types of treatment, NED patients were significantly more likely to receive chemotherapy (OR 7.05, 90% CI 3.17–15.69, *p* < 0.001), more likely to be enrolled in a clinical trial (OR 5.55, 90% CI 5.55–12.50, *p* < 0.001), and had an increased likelihood of having surgery as well (OR 2.42, 90% CI 1.12–5.26, *p* = 0.061).

Patients in the ED cohort were also significantly less likely to receive any therapy compared to NED patients among those with CNS tumors (*p* = 0.008); this finding was also statistically significant within the subgroups of surgery, chemotherapy, and radiation (*p* = 0.032; *p* = 0.002; and *p* = 0.033 respectively). There were no significant differences when comparing treatment and subtypes of treatment between ED and NED (patients within hematologic malignancy and solid tumor patients).

### 3.2 Time course of presentation

**FIGURE 4:**
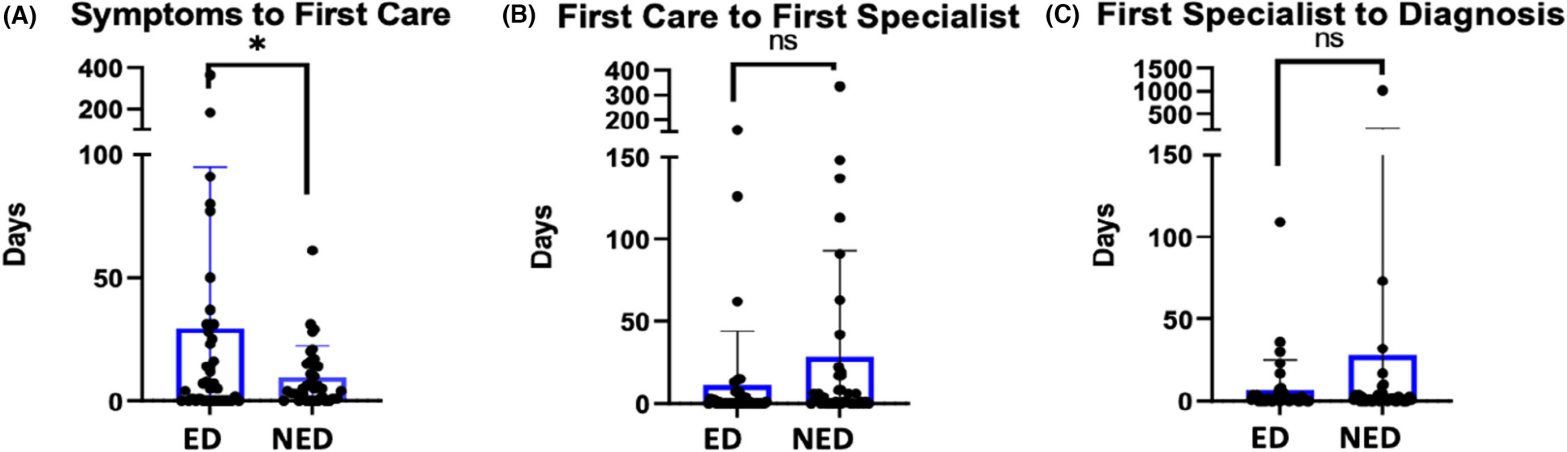
Time course of presentation for early death (ED) cohort versus non‐early death (NED). Each patient is represented by a dot with IQR identified with blue box and SEM noted with error bars. ED and NED groups were compared using unpaired *t*‐tests with Welch's correction. (A) represents days from symptom onset to first seeking care. (B) represents days from first seeking care to first seeing a specialist. (C) represents days from first seeing a specialist to diagnosis. Statistical significance is marked as ns for not significant, * for *p* < 0.1.

Time course was evaluated as time from symptom onset to seeking care, from first care to first specialist visit, from first specialist visit to diagnosis, and from first symptom to diagnosis using an unpaired *t*‐test with Welch's correction (Figure 4). Using a significance level of 0.10, time from symptom onset to first seeking care was significantly greater in the ED group versus NED group (mean 29.4 days vs. 9.8 days, *p* = 0.07). Mean time from first seeking care to first specialist visit was 12.2 days in the ED cohort as compared to 28.4 days in the NED group (*p* = 0.15). Time from first specialist to diagnosis was also non‐significantly shorter in the ED cohort with a mean of 6.7 days in the ED cohort as compared to 28.8 days in the NED group (*p* = 0.39). Lastly, mean time from symptom onset to diagnosis was 55 days in the ED group and 113 days in the NED group (*p* = 0.13).

### 3.3 Cause of death

**FIGURE 5:**
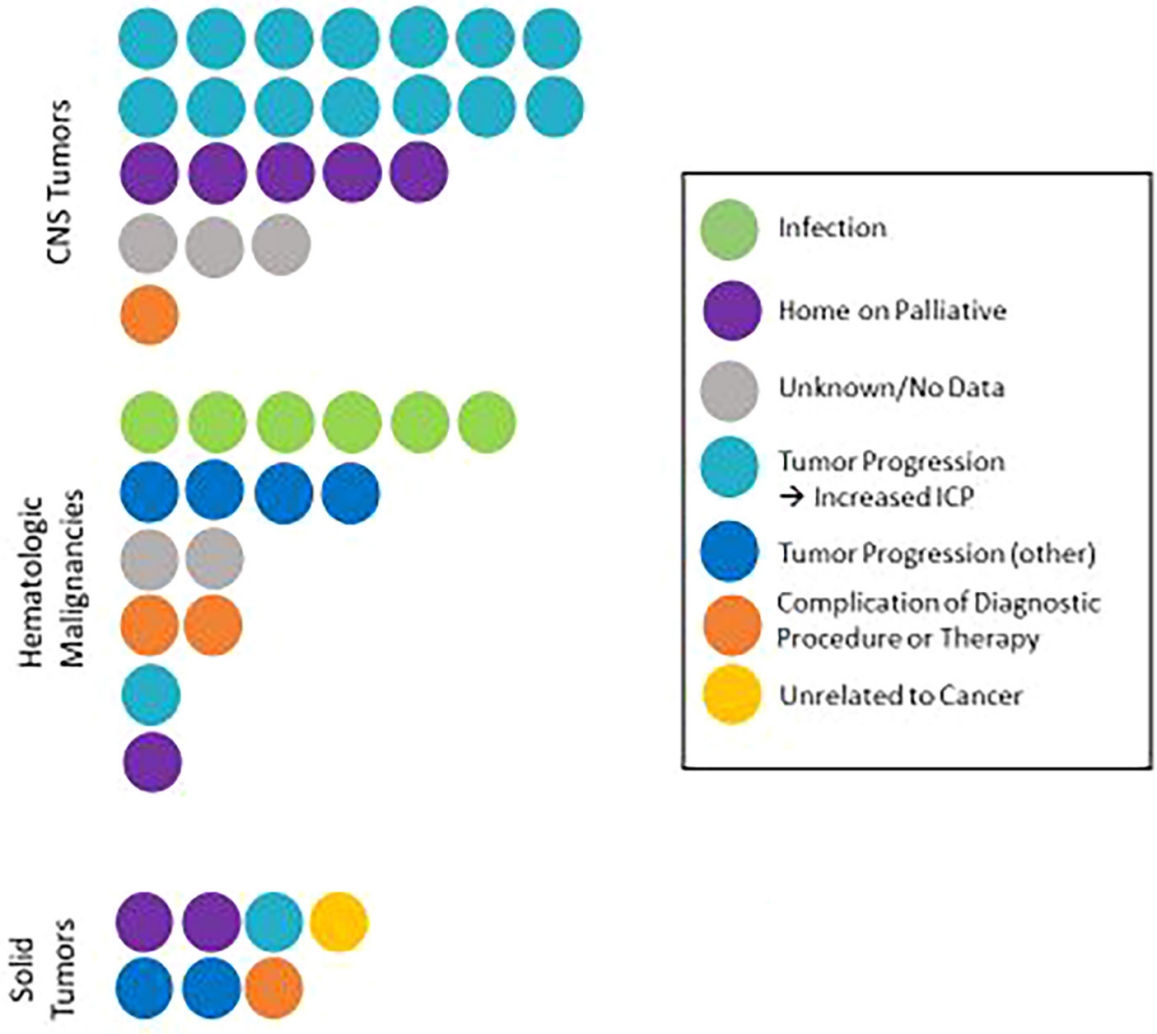
Cause of death. For the early death cohort, causes of death as identified by chart review and confirmed with state death certificate data are demonstrated by each disease subgroup.

Among the patients in the ED cohort, cause of death was grouped into infection, tumor progression leading to increased intracranial pressure, other tumor progression, complication of diagnostic procedure or therapy, home on palliative care with subsequent death, death unrelated to oncologic diagnosis, or unknown (no data available). This was sub‐grouped by disease category (Figure 5). In the brain/CNS ED cohort, the most common causes of death were tumor progression resulting in increased ICP (60.9%) and 21.7% of patients died after going home with palliative care. Among patients with hematologic malignancies, the most common cause of death was infection (37.5%) followed by tumor progression (25%). In the ED cohort of patients with solid tumors, 28.6% of patients went home with palliative care and 28.6% of patients died from tumor progression. For each of the patients in the ED cohort, a brief description of their diagnostic course and cause of death can be found in Table S2.

## 4 DISCUSSION

We conducted a single‐center, patient‐level, retrospective cohort study to better understand the population of children who die within 30 days of an oncologic diagnosis. We identified a significantly increased risk of ED among children with brain/CNS tumors and, within that group, children with ATRT and HGG. Children in the ED cohort were significantly less likely to receive any form of treatment, especially chemotherapy, and were significantly less likely to be enrolled on clinical trials. The decreased likelihood of enrollment on clinical trials has previously been reported completed on the database level.^2^, ^13^ ED and NED patients' symptoms at presentation were similar when comparing all patients and within disease groups. A short narrative of cause of death on each patient is available in the supplemental table as well.

The most unique finding of this study was the prolonged time course from symptom onset to first seeking care in the ED group. Previous work has demonstrated that advanced disease at diagnosis confers increased risk of ED, which has led to the hypothesis that perhaps more aggressive disease is the underlying problem.^2^ The ED cohort, in general, was often quite ill at presentation, and thus this delay in presentation was surprising. Once patients first sought care, there was no significant difference between ED and NED patients in time to receiving specialist care and receiving a diagnosis. Prior literature has demonstrated that shorter time to diagnosis can have improve prognosis in some childhood cancers, such as melanoma and retinoblastoma.^14^, ^15^, ^16^ There are specific groups known to be at risk for diagnostic delays including adolescents, children whose parents have lower education levels, less health literacy, no formal employment, and rural residence, as well as patients with specific diseases, such as renal and brain tumors.^17^, ^18^ Disparity in both diagnosis and survival by race/ethnic group as well as socioeconomic status have further been demonstrated within specific pediatric cancers, such as bone tumors, leukemia, lymphoma, and extracranial solid tumors.^19^, ^20^ Diagnostic delays for these groups have been well documented globally, but this is the first study to connect diagnostic delays to ED in childhood cancer. Future work is needed to understand the specific causes of these delays in initial presentation so that we may be able to address this risk factor.

When evaluating cause of death, children with CNS tumors were most likely to die from in‐hospital disease progression resulting in increased ICP or, after receiving their poor prognosis, went home on palliative care, which aligns with prior research demonstrating very few pediatric patients with CNS tumors die from treatment‐related mortality.^21^ Infection was a much more common cause of death in the hematologic malignancies group, while the patients in the ED cohort with solid tumors had a greater heterogeneity in cause of death. It is unclear if a lack of access to novel trials for diseases such as high‐grade glioma was a contributing factor to the number of patients in the CNS tumor group who went home on palliative care. This finding is supported by prior work demonstrating decreased trial involvement over time, particularly among adolescents and those with CNS tumors.^22^ We expect that, given recent advancements in options for children who previously had few or no treatment options, this number will decrease over time. Within the hematologic malignancies ED group, the most common cause of death was infection, which has been reported in hematologic malignancies previously.^23^, ^24^ As supportive care and infectious prophylaxis has improved over time,^25^, ^26^ we anticipate that ED rates in this group may improve as well.

Despite EMR review, we were unable to determine if genetic cancer predisposition syndromes contributed to the ED cohort, as none of the patients under 1 year of age with tumor types that were highly concerning for underlying genetic abnormality received these evaluations. While evaluation of a pathogenic germline variant may not have prevented these specific patients' ED, there remains a significant impact that this information could have on these patients' family members. Given that there is evidence for improved survival with early detection in many hereditary cancer syndromes, genetic counseling should still be considered for these families after the death of their child, even if the interaction with the family was brief.^27^, ^28^

Large population databases, such as the SEER database, provide the opportunity for a global overview but cannot provide details such as familial structure, access to primary care, initial symptoms, barriers and delays in diagnosis and treatment, and individual timelines from symptom onset through diagnosis, therapy, and outcome. Manual chart review is a time‐intensive process that is challenging to complete thoroughly on a large scale. By utilizing EMR review, though, we were able to gain some unique insight, particularly into time course of symptoms, cause of death, and patient‐level socioeconomic risk factors. However, here remained variables that we hoped to better understand that were not uniformly described in the EMR despite manual review. Some of these variables that were rarely mentioned included parental employment status, health insurance status at time of diagnosis, family income level, barriers to care noted by parents and medical teams, and access to primary care prior to diagnosis. Other limitations of this study include the small sample size, single‐institutional nature, long period of study (given that there have been significant changes in diagnostic, therapeutic, and supportive care during the time course of this cohort), and the limitation of data to what was available by chart review and death certificates.

This study is the first to attempt to better characterize children who die shortly after an oncologic diagnosis using patient medical‐record level risk factors. By utilizing patient‐level data from our EMR, we were able to obtain more data on the time course of illness, details regarding how they presented and were diagnosed, and understanding of circumstances and causes of death. These findings provide unique insight into the ED population, but questions remain, including the perspective of families and their treating teams regarding barriers to care they faced. While this manual chart review adds to existing literature in regard to its individualized review of time course to symptoms and review of the narrative course of each patient leading to their death, we were unable to reliably identify information in most charts regarding parental health literacy, access to transportation and primary medical care prior to diagnosis, health insurance status prior to diagnosis, family structure at time of diagnosis, and perceived barriers to care and diagnosis. Further evaluations into the social, economic, and medical barriers that may contribute to childhood cancer ED are essential. Obtaining more family‐specific socioeconomic information requires a prospective intervention using direct patient interviews. Accordingly, we have now opened an acceptability trial whereby we will interview families whose children died within 30 days of a cancer diagnosis so that we may be able to determine whether this methodology will allow us to answer these questions. Once we can better understand this unfortunate group of patients, we may be able to design and implement interventions to reduce the frequency of ED in childhood cancer.

## AUTHOR CONTRIBUTIONS

**Katherine T. Lind:** Data curation (lead); formal analysis (equal); investigation (equal); writing – original draft (lead). **Elizabeth Molina:** Formal analysis (supporting); methodology (supporting); writing – review and editing (equal). **Amy Mellies:** Formal analysis (equal); methodology (equal); writing – review and editing (supporting). **Kami Wolfe Schneider:** Data curation (supporting); writing – review and editing (supporting). **William Daley:** Resources (equal); writing – review and editing (equal). **Adam L. Green:** Conceptualization (lead); data curation (supporting); formal analysis (supporting); funding acquisition (lead); investigation (equal); methodology (equal); project administration (lead); supervision (lead); writing – review and editing (lead).

## FUNDING INFORMATION

Statistical support was funded by NCI Cancer Center Support Grant (P30AC046934). Other funding was provided by the Morgan Adams Foundation and the University of Colorado Cancer Center Cancer Prevention and Control Program.

## CONFLICT OF INTEREST STATEMENT

The authors have no conflicts of interest to disclose.

## PRECIS

Death within 30 days of a cancer diagnosis, termed early death (ED), is a poorly understood phenomenon in pediatric oncology and comprises 7.5% of all pediatric cancer deaths. In this first medical record‐level analysis of ED, we surprisingly found that patients with ED had a longer time from symptom onset to care seeking and also identified that cause of death differed by cancer category; we highlight the need for future prospective studies.

## Supporting information


Tables S1–S2.

## Data Availability

All de‐identified data from this project is included in the manuscript and supplemental tables.
